# What can we learn from Plausible Values?

**DOI:** 10.1007/s11336-016-9497-x

**Published:** 2016-04-06

**Authors:** Maarten Marsman, Gunter Maris, Timo Bechger, Cees Glas

**Affiliations:** Department of Psychology, University of Amsterdam, Nieuwe Prinsengracht 129-B, P.O. Box 15906, 1001 NK Amsterdam, The Netherlands; Cito, Schwaig, Germany; University of Twente, Enschede, The Netherlands

**Keywords:** plausible values, item response theory, educational surveys, Bayesian theory

## Abstract

In this paper, we show that the marginal distribution of plausible values is a consistent estimator of the true latent variable distribution, and, furthermore, that convergence is *monotone* in an embedding in which the number of items tends to infinity. We use this result to clarify some of the misconceptions that exist about plausible values, and also show how they can be used in the analyses of educational surveys.

## Introduction

In educational surveys, an *item response theory* (IRT) model is used to model the conditional distribution of a vector of item responses $\mathbf {X} = \{X_1\text {, }X_2\text {, }\ldots \text {, }X_n\}$ as a function of a latent random variable (ability) ${\Theta }$, where the item response functions are monotonically increasing in ability. The IRT model characterizes the latent variable $\Theta$, and the goal of educational surveys is to estimate the distribution of $\Theta$ which we denote by *f*. Together, the IRT model and the ability distribution induce the following statistical model:

$$\begin{aligned} P(\mathbf {X}_f=\mathbf {x}) = \int _{\mathbb {R}}P(\mathbf {X}=\mathbf {x} \mid \theta )f(\theta ) \text {d}\theta , \end{aligned}$$

where $P(\mathbf {X}_f)$ is the true data distribution of which we obtain a sample. Throughout this paper, we assume that the IRT model is given, and focus on the unknown *f*. We consider the usual case where the item responses $X_i$ are discrete with a finite number of possible realizations but note that the results remain the same when the $X_i$ are continuous and sums are replaced by integrals.

There are four possible approaches to estimate *f* from the observed data. The first entails the use of a function *T* such that $T(\mathbf {X}) \sim \Theta$. If $\mathbf {X}$ is discrete, realizations of $T(\mathbf {X})$ are discrete as well. The second approach requires a function *T* such that $T(\mathbf {X}) \overset{\mathcal {L}}{\longrightarrow }\Theta$, i.e., a random variable that, asymptotically, has the same distribution as $\Theta$. This can be any *T* that is a consistent estimator of $\Theta$ such as the Maximum Likelihood (ML) or Weighted ML (WML) estimator (Warm, 1989). The third approach is to use the data to generate a random variable $\Theta ^*$ such that  and $\Theta ^*\sim \Theta$. By definition, $\Theta$ and $\Theta ^*$ are *exchangeable* and their joint density can be written as follows:

1 $$\begin{aligned} f(\theta ^*,\theta ) = \sum _{\mathbf {x}}f(\theta ^*\mid \mathbf {X}=\mathbf {x})f(\theta \mid \mathbf {X}=\mathbf {x})P(\mathbf {X}_f=\mathbf {x}), \end{aligned}$$

where summation is over all possible realizations of $\mathbf {X}$. The conditional distributions $f(\theta \mid \mathbf {X})$ are posterior distributions and it easily follows that the marginal distribution of draws from these posteriors equals the population distribution. Thus, if we sample from the correct posteriors, the population distribution can be recovered in a straightforward way. The problem, however, is that we do not know the correct posterior because we do not know *f*. In practice, we would therefore use a prior distribution^1^*g* to generate random variables $\tilde{\Theta } \mid \mathbf {X}$ (i.e., sample from the posteriors $g(\theta \mid \mathbf {X})$). The random variables $\tilde{\Theta } \mid \mathbf {X}$ are called *plausible values* (PVs) in the psychometric literature (Mislevy, 1991; Mislevy, Beaton, Kaplan, & Sheehan, 1993). Using PVs to estimate *f* constitutes the fourth and final approach and the one this paper is about.

In this paper, we prove that under mild regularity conditions, PVs are random variables of the form $\tilde{\Theta }\mid \mathbf {X}$ such that $\tilde{\Theta } \overset{\mathcal {L}}{\longrightarrow }\Theta$. That is, we will show that the marginal distribution of the PVs is a consistent estimator of *f*. More specifically, let

$$\begin{aligned} \tilde{g}(\theta ) = \sum _{\mathbf {x}} g(\theta \mid \mathbf {X}=\mathbf {x})P(\mathbf {X}_f=\mathbf {x}) \end{aligned}$$

denote the marginal distribution of the PVs.

This distribution is intractable but easily sampled from; that is, nature provides realizations from $P(\mathbf {X}_f)$, which we then use to sample PVs^2^.

It is well known that the *empirical cumulative distribution function (ecdf)* of the PVs is a consistent estimator of $\tilde{g}$ as the number of persons goes to infinity. Our main goal is to demonstrate that $\tilde{g}$ in turn converges in law to *f* (i.e., $\tilde{\Theta } = \Theta _{\tilde{g}} \overset{\mathcal {L}}{\longrightarrow }\Theta _f$) as the number of items goes to infinity. The following example gives a foretaste of what this paper is about.

### *Example 1*

**Fig. 1:**
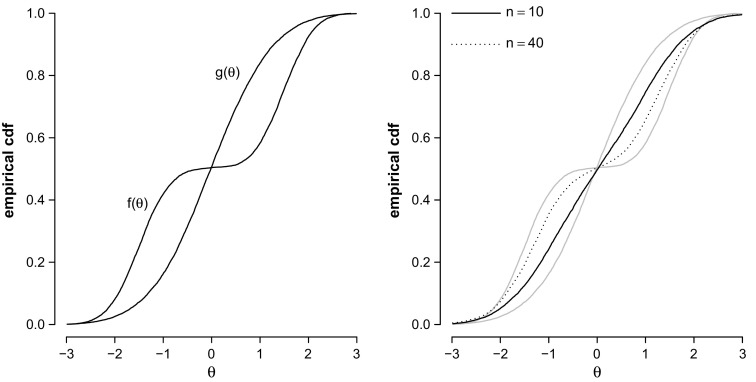
Ecdfs of $N =$ 10,000 draws from $f(\theta )$ and $N =$ 10,000 draws from the standard normal prior distribution $g(\theta )$ are shown in both panels (in *gray* in the *right panel*). Ecdfs of the marginal distributions of PVs are shown in the *right panel*.

We generate responses of $$N = $$ 10,000 persons on a test consisting of *n* Rasch items with difficulty parameters sampled uniformly between $$-1$$ and 1. The ability distribution *f* is a mixture with two normal components whose ecdf is shown in the left panel of Fig. 1. One component may, for instance, be the distribution for the boys and the other one is that for the girls.

The analyst is unaware of the difference between the boys and the girls and chooses *g* to be a standard normal distribution. We now generate a single PV for each of the *N* persons; once for a test with $n = 10$ items and once for a test with $n = 40$ items. The PV distributions are shown in the right panel of Fig. 1. Figure 1 shows that the distribution of the PVs is not the standard normal. In fact, with 40 items, it begins to resemble the true ability distribution even though the population model is clearly wrong.

Instead of proving that $\tilde{g}$ converges in law to *f*, we will prove a stronger result. Namely, that $\tilde{g}$ converges to *f* in *Expected Kullback-Leibler (EKL) divergence* (Kullback & Leibler, 1951) as the number of items *n* tends to infinity.

### **Definition**

The Expected (posterior) Kullback-Leibler (EKL) divergence between $\Theta _f \mid \mathbf {X}$ and $\Theta _g\mid \mathbf {X}$, w.r.t. $f(\Theta \mid \mathbf {X})$ and $P(\mathbf {X}_f)$ is

$$\begin{aligned} \mathbb {E}(\Delta (\Theta _f{\text { ; }}\Theta _g \mid \mathbf {X}_f))&= \sum _{\mathbf {x}}\Delta (\Theta _f{\text { ; }}\Theta _g \mid \mathbf {X}=\mathbf {x})P(\mathbf {X}_f=\mathbf {x})\\&=\sum _{\mathbf {x}} \left[ \int _\mathbb {R} \ln \left( \frac{f(\theta \mid \mathbf {X}=\mathbf {x})}{g(\theta \mid \mathbf {X}= \mathbf {x})}\right) f(\theta \mid \mathbf {X}=\mathbf {x}) {\text {d}} \theta \right] P(\mathbf {X}_f=\mathbf {x}), \end{aligned}$$

where $\Delta (\Theta _f\text { ; }\Theta _g\mid \mathbf {X})$ denotes the Kullback-Leibler (KL) divergence of $f(\Theta \mid \mathbf {X})$ and $g(\Theta \mid \mathbf {X})$ with respect to $f(\Theta \mid \mathbf {X})$, with $0\ln (0)\equiv 0$.

Throughout this paper, we assume that all divergences are finite, which is true if the support of *g* contains that of *f* (i.e., *f* is absolutely continuous w.r.t. *g*) *almost everywhere (a.e.)*. Note that the KL and EKL divergences that we use in this paper are non-symmetric in their arguments, yet their values are always non-negative and zero if and only if the compared probability distributions are the same *a.e.* (see Theorem 9.6.1 in Cover & Thomas, 1991, p. 232).

We demonstrate in the next section that convergence in EKL divergence is indeed stronger than convergence in law. Then, we prove that EKL divergence is monotonically non-increasing in *n* and tends to zero as the number of items *n* tends to infinity: Informally, this means that $\tilde{g}$ will always get closer to *f* as *n* grows, as we saw in the example. Having thus established our main result, we discuss a number of implications for educational surveys and show that quite a lot can be learned from PVs. Throughout, PISA data will be used for illustration. The paper ends with a discussion.

## Convergence in EKL divergence implies convergence in law

To demonstrate that $\tilde{g}$ converges in law to *f*, it is sufficient to prove that $\tilde{g}$ converges to *f* in KL divergence as this implies convergence in law (DasGupta, 2008, p. 21). The following theorem implies that convergence in EKL divergence is stronger than convergence in KL divergence.

### **Theorem 1**

Given an IRT model $P(\mathbf {X} \mid \theta )$ and assuming that the support of *g* contains the support of *f*, the KL divergence of $\Theta _{\tilde{g}}$ w.r.t. $\Theta _{f}$, i.e.,

$$\begin{aligned} \Delta (\Theta _f{\text { ; }}\Theta _{\tilde{g}}) = \int _{\mathbb {R}} \ln \frac{f(\theta )}{\tilde{g}(\theta )}f(\theta ){\text {d}}\theta , \end{aligned}$$

is always smaller than or equal to EKL divergence. That is,

$$\begin{aligned} \Delta (\Theta _f{\text { ; }}\Theta _{\tilde{g}}) \le \mathbb {E}(\Delta (\Theta _f{\text { ; }}\Theta _{g} \mid \mathbf {X}_f)). \end{aligned}$$

### *Proof*

We start with rewriting the logarithm of the ratio of $\tilde{g}$ over *f*

$$\begin{aligned} \ln \frac{\tilde{g}(\theta )}{f(\theta )}&= \ln \left\{ \frac{\sum _{\mathbf {x}}g(\theta \mid \mathbf {X}=\mathbf {x})P(\mathbf {X}_f=\mathbf {x})}{\sum _{\mathbf {x}}f(\theta \mid \mathbf {X}=\mathbf {x})P(\mathbf {X}_f=\mathbf {x})}\right\} \\&=\ln \left\{ \sum _{\mathbf {x}}\frac{g(\theta \mid \mathbf {X}=\mathbf {x})P(\mathbf {X}_f=\mathbf {x})}{f(\theta \mid \mathbf {X}=\mathbf {x})P(\mathbf {X}_f=\mathbf {x})} \frac{f(\theta \mid \mathbf {X}=\mathbf {x})P(\mathbf {X}_f=\mathbf {x})}{\sum _{\mathbf {x}}f(\theta \mid \mathbf {X}=\mathbf {x})P(\mathbf {X}_f=\mathbf {x})}\right\} \\&=\ln \left\{ \sum _{\mathbf {x}}\frac{g(\theta \mid \mathbf {X}=\mathbf {x})}{f(\theta \mid \mathbf {X}=\mathbf {x})} P(\mathbf {X}=\mathbf {x} \mid \theta )\right\} \\&\ge \sum _{\mathbf {x}} \ln \frac{g(\theta \mid \mathbf {X}=\mathbf {x})}{f(\theta \mid \mathbf {X}=\mathbf {x})} P(\mathbf {X}=\mathbf {x} \mid \theta ), \end{aligned}$$

using Jensen’s inequality. Thus, we obtain

$$\begin{aligned} \ln \frac{f(\theta )}{\tilde{g}(\theta )}&\le \sum _{\mathbf {x}} \ln \frac{f(\theta \mid \mathbf {X}=\mathbf {x})}{g(\theta \mid \mathbf {X}=\mathbf {x})} P(\mathbf {X}=\mathbf {x} \mid \theta ). \end{aligned}$$

Integrating both sides of this expression w.r.t. *f* gives the desired result:

$$\begin{aligned} \int _{\mathbb {R}}\ln \frac{f(\theta )}{\tilde{g}(\theta )} f(\theta ) \text {d} \theta&\le \int _{\mathbb {R}}\sum _{\mathbf {x}} \ln \frac{f(\theta \mid \mathbf {X}=\mathbf {x})}{g(\theta \mid \mathbf {X}=\mathbf {x})} P(\mathbf {X}=\mathbf {x} \mid \theta ) f(\theta ) \text {d}\theta \\&= \sum _{\mathbf {x}}\int _{\mathbb {R}} \ln \frac{f(\theta \mid \mathbf {X}=\mathbf {x})}{g(\theta \mid \mathbf {X}=\mathbf {x})} f(\theta \mid \mathbf {X}=\mathbf {x}) \text {d}\theta \text { }P(\mathbf {X}_f=\mathbf {x}). \end{aligned}$$

It follows that $\tilde{g}$ converges in law to *f* if $\tilde{g}$ converges to *f* in EKL. Proving convergence in EKL will be the burden of the ensuing sections.$\square$

## Monotone Convergence of Plausible Values

Before we can state our first result in Theorem 2, we need two Lemma’s.

### **Lemma 1**

Given an IRT model $P(\mathbf {X} \mid \theta )$ and assuming that the support of *g* contains the support of *f*, the EKL divergence of $\Theta _f \mid \mathbf {X}$ and $\Theta _g \mid \mathbf {X}$, w.r.t. $f(\Theta \mid \mathbf {X})$ and $P(\mathbf {X}_f)$, equals prior divergence minus marginal divergence, that is,

$$\begin{aligned} \mathbb {E}(\Delta (\Theta _f{\text { ; }}\Theta _g\mid \mathbf {X}_f))=\Delta (\Theta _f{\text { ; }}\Theta _g) - \Delta (\mathbf {X}_f{\text { ; }}\mathbf {X}_g). \end{aligned}$$

### *Proof*

Using the definition of the posterior, and given the IRT model $P(\mathbf {X}\mid \theta )$, we rewrite the EKL divergence as follows:

$$\begin{aligned} \mathbb {E}(\Delta (\Theta _f\text { ; }\Theta _g \mid \mathbf {X}_f)) =&\sum _{\mathbf {x}} \int _{\mathbb {R}} \ln \left( \frac{\frac{P(\mathbf {X}=\mathbf {x} \mid \theta )f(\theta )}{P(\mathbf {X}_f=\mathbf {x})}}{\frac{P(\mathbf {X}=\mathbf {x} \mid \theta )g(\theta )}{P(\mathbf {X}_g=\mathbf {x})}}\right) f(\theta \mid \mathbf {X}=\mathbf {x})\text {d}\theta \text { }P(\mathbf {X}_f=\mathbf {x})\\ =&\sum _{\mathbf {x}} \int _{\mathbb {R}} \ln \left( \frac{f(\theta )}{g(\theta )} \frac{P(\mathbf {X}_g=\mathbf {x})}{P(\mathbf {X}_f=\mathbf {x})}\right) f(\theta \mid \mathbf {X}=\mathbf {x})\text {d}\theta \text { }P(\mathbf {X}_f=\mathbf {x}), \end{aligned}$$

where $P(\mathbf {X}_g)$ is the distribution of the data under the prior *g*. Using properties of the logarithm, we obtain

$$\begin{aligned} \mathbb {E}(\Delta (\Theta _f\text { ; }\Theta _g \mid \mathbf {X}_f)) =&\sum _{\mathbf {x}} \int _{\mathbb {R}} \ln \left( \frac{f(\theta )}{g(\theta )}\right) f(\theta \mid \mathbf {X}=\mathbf {x})\text {d}\theta \text { }P(\mathbf {X}_f=\mathbf {x})\\&+ \sum _{\mathbf {x}} \int _{\mathbb {R}} \ln \left( \frac{P(\mathbf {X}_g=\mathbf {x})}{P(\mathbf {X}_f=\mathbf {x})}\right) f(\theta \mid \mathbf {X}=\mathbf {x})\text {d}\theta \text { }P(\mathbf {X}_f=\mathbf {x}). \end{aligned}$$

If we sum over the possible values of $\mathbf {X}$ in the first term and integrate over $\Theta$ in the second term, respectively, we obtain

$$\begin{aligned} \mathbb {E}(\Delta (\Theta _f\text { ; }\Theta _g \mid \mathbf {X}_f))&= \int _{\mathbb {R}} \ln \left( \frac{f(\theta )}{g(\theta )}\right) f(\theta )\text {d}\theta + \sum _{\mathbf {x}}\ln \left( \frac{P(\mathbf {X}_g=\mathbf {x})}{P(\mathbf {X}_f=\mathbf {x})}\right) P(\mathbf {X}_f=\mathbf {x})\\&= \int _{\mathbb {R}} \ln \left( \frac{f(\theta )}{g(\theta )}\right) f(\theta )\text {d}\theta - \sum _{\mathbf {x}}\ln \left( \frac{P(\mathbf {X}_f=\mathbf {x})}{P(\mathbf {X}_g=\mathbf {x})}\right) P(\mathbf {X}_f=\mathbf {x})\\&=\Delta (\Theta _f\text { ; }\Theta _g) - \Delta (\mathbf {X}_f\text { ; }\mathbf {X}_g). \end{aligned}$$

It follows that EKL divergence of the posterior distribution is equal to the difference between *prior divergence*$\Delta (\Theta _f\text { ; }\Theta _g)$ and *marginal divergence*$\Delta (\mathbf {X}_f\text { ; }\mathbf {X}_g)$ (i.e., divergence of $P(\mathbf {X}_g)$ w.r.t. $P(\mathbf {X}_f)$).$\square$

Lemma 1 implies that $\mathbb {E}(\Delta (\Theta _f\text { ; }\Theta _g\mid \mathbf {X}_f))$ equals zero if and only if prior divergence is equal to marginal divergence. Since the divergences are finite and non-negative, we find that

$$\begin{aligned} \Delta (\Theta _f\text { ; }\Theta _g) \ge \Delta (\mathbf {X}_f\text { ; }\mathbf {X}_g). \end{aligned}$$

We will now prove that $\Delta (\mathbf {X}_f\text { ; }\mathbf {X}_g)$ is a monotone non-decreasing sequence in the number of items *n* with $\Delta (\Theta _f\text { ; }\Theta _g)$ as an upper bound. To this aim, we consider what happens to marginal divergence when an item is added ( i.e., *n* is increased to $n+1$). To fix the notation, let $X_1\text {, } X_2\text {, } ...$ denote an infinite sequence of item responses, with $X_n$ the *n*-th element and $\mathbf {X}_n$ a vector consisting of the first *n* elements of this sequence.

### **Lemma 2**

Given an IRT model $P(\mathbf {X} \mid \theta )$ and assuming that the support of *g* contains the support of *f*, the marginal divergence for $n+1$ observations is larger than or equal to marginal divergence for *n* observations:

$$\begin{aligned} \Delta (\mathbf {X}_{f,n+1}; \mathbf {X}_{g,n+1}) \ge \Delta (\mathbf {X}_{f,n}; \mathbf {X}_{g,n}). \end{aligned}$$

### *Proof*

The marginal divergence for $n+1$ items is

$$\begin{aligned} \Delta (\mathbf {X}_{f,n+1}\text { ; }\mathbf {X}_{g,n+1}) = \sum _{\mathbf {x}_{n+1} }\ln \left( \frac{P(\mathbf {X}_{f,n+1} =\mathbf {x}_{n+1})}{P(\mathbf {X}_{g,n+1} =\mathbf {x}_{n+1})}\right) P(\mathbf {X}_{f,n+1} =\mathbf {x}_{n+1}). \end{aligned}$$

Conditioning on the first *n* observations and factoring the distribution, we obtain

$$\begin{aligned} \Delta (\mathbf {X}_{f,n+1}\text { ; }\mathbf {X}_{g,n+1}) =&\sum _{\mathbf {x}_{n}}\sum _{x_{n+1}} \ln \left( \frac{P(X_{f,n+1}=x_{n+1} \mid \mathbf {X}_n=\mathbf {x}_n)}{P(X_{g,n+1}=x_{n+1}\mid \mathbf {X}_n=\mathbf {x}_n)} \frac{P(\mathbf {X}_{f,n}= \mathbf {x}_n)}{P(\mathbf {X}_{g,n}=\mathbf {x}_n)}\right) \\&\times P(X_{f,n+1}=x_{n+1}\mid \mathbf {X}_n=\mathbf {x}_n)P(\mathbf {X}_{f,n}=\mathbf {x}_n). \end{aligned}$$

This is equal to

$$\begin{aligned} \Delta (\mathbf {X}_{f,n+1}\text { ; }\mathbf {X}_{g,n+1})&= \sum _{\mathbf {x}_{n}} \sum _{x_{n+1}} \ln \left( \frac{P(X_{f,n+1}=x_{n+1}\mid \mathbf {X}_n=\mathbf {x}_n)}{P(X_{g,n+1}=x_{n+1}\mid \mathbf {X}_n=\mathbf {x}_n)}\right) \\&\quad \times P(X_{f,n+1}=x_{n+1}\mid \mathbf {X}_n=\mathbf {x}_n)P(\mathbf {X}_{f,n}=\mathbf {x}_n)\\&\qquad + \sum _{\mathbf {x}_{n}} \ln \left( \frac{P(\mathbf {X}_{f,n}=\mathbf {x}_n)}{P(\mathbf {X}_{g,n}=\mathbf {x}_n)}\right) P(\mathbf {X}_{f,n}=\mathbf {x}_n)\\&= \mathbb {E}(\Delta (X_{f,n+1}\text { ; }X_{g,n+1}|\mathbf {X}_{f,n})) + \Delta (\mathbf {X}_{f,n}\text { ; }\mathbf {X}_{g,n}), \end{aligned}$$

a result closely related to the *chain rule* of KL divergence (Cover & Thomas, 1991, p. 23). Since $\mathbb {E}(\Delta (X_{f,n+1}\text { ; }X_{g,n+1}\mid \mathbf {X}_{f,n})) \ge 0$, we see that

$$\begin{aligned} \Delta (\mathbf {X}_{f,n+1}\text { ; }\mathbf {X}_{g,n+1}) \ge \Delta (\mathbf {X}_{f,n}\text { ; }\mathbf {X}_{g,n}). \end{aligned}$$

$$\square $$

Using Lemmas 1 and 2, we can now state Theorem 2.

### **Theorem 2**

(Monotonicity Theorem) Given an IRT model $P(\mathbf {X} \mid \theta )$ and assuming that the support of *g* contains the support of *f*, $\mathbb {E}(\Delta (\Theta _f{\text { ; }}\Theta _g\mid \mathbf {X}_{f,n}))$ is monotone non-increasing in the number of items *n*.

### *Proof*

From Lemmas 1 and 2, we obtain

$$\begin{aligned} \mathbb {E}(\Delta (\Theta _f\text { ; }\Theta _g\mid \mathbf {X}_{f,n+1})) = \Delta (\Theta _f\text { ; }\Theta _g)&- \Delta (\mathbf {X}_{f,n+1}\text { ; }\mathbf {X}_{g,n+1})\\ = \Delta (\Theta _f\text { ; }\Theta _g)&- \mathbb {E}(\Delta (X_{f,n+1}\text { ; }X_{g,n+1} \mid \mathbf {X}_{f,n}))\\&- \Delta (\mathbf {X}_{f,n}\text { ; }\mathbf {X}_{g,n}), \end{aligned}$$

and Lemma 1 shows that the difference of the first and the last terms is equal to the EKL divergence for *n* items. Thus, we have

$$\begin{aligned} \mathbb {E}(\Delta (\Theta _f\text { ; }\Theta _g \mid \mathbf {X}_{f,n+1})) = \mathbb {E}(\Delta (\Theta _f\text { ; }\Theta _g \mid \mathbf {X}_{f,n})) - \mathbb {E}(\Delta (X_{f,n+1}\text { ; }X_{g,n+1} \mid \mathbf {X}_{f,n})). \end{aligned}$$

This implies a sequence of EKL divergences which adheres to the (in-)equality:

$$\begin{aligned} 0 \le \mathbb {E}(\Delta (\Theta _f\text { ; }\Theta _g \mid \mathbf {X}_{f,n+1})) \le \mathbb {E}(\Delta (\Theta _f\text { ; }\Theta _g \mid \mathbf {X}_{f,n})) \le \Delta (\Theta _f\text { ; }\Theta _g), \end{aligned}$$

i.e., a monotone non-increasing sequence in *n* with lower bound 0. Since prior divergence is finite by assumption, it is an upper bound for this sequence. $\square$

## Large Sample Properties of Plausible Values

The Monotonicity Theorem shows that the sequence of EKL divergences converges in an embedding in which $n \rightarrow \infty$. This does not imply that the marginal distribution of PVs converges to *f*, since the sequence of EKL divergences may converge to a number that is strictly larger than zero. We have yet to show that the sequence of EKL divergences converges to zero. Since by Lemma 1 the EKL divergence is equal to the difference between prior and marginal divergence, we may equivalently show that the inequality

2 $$\begin{aligned} \Delta (\Theta _f\text { ; }\Theta _g) \ge \Delta (\mathbf {X}_{f,n}\text { ; }\mathbf {X}_{g,n}) \end{aligned}$$

becomes an equality as $n \rightarrow \infty$.

### **Theorem 3**

(Convergence Theorem) Given an IRT model $P(\mathbf {X} \mid \theta )$ and assuming that the support of *g* contains the support of *f*,

$$\begin{aligned} \lim _{n\rightarrow \infty } \mathbb {E}(\Delta (\Theta _f{\text { ; }}\Theta _g\mid \mathbf {X}_{f,n})) = 0 \end{aligned}$$

if the sequence of posteriors converges to a degenerate distribution.

### *Proof*

We start with a direct proof of (2) (suppressing the dependence on *n*). Note first that,

3 $$\begin{aligned} \forall \mathbf {x}: \ln \frac{P(\mathbf {X}_f=\mathbf {x})}{P(\mathbf {X}_g=\mathbf {x})}&= -\ln \frac{P(\mathbf {X}_g=\mathbf {x})}{P(\mathbf {X}_f=\mathbf {x})}\nonumber \\&= -\ln \frac{\int _\mathbb {R} P(\mathbf {X}=\mathbf {x} \mid \theta )g(\theta )\text {d}\theta }{\int _\mathbb {R} P(\mathbf {X}=\mathbf {x}\mid \theta )f(\theta )\text {d}\theta }\nonumber \\&= -\ln \int _\mathbb {R}\frac{P(\mathbf {X}=\mathbf {x}\mid \theta )g(\theta ) }{P(\mathbf {X}=\mathbf {x}\mid \theta )f(\theta )} \frac{P(\mathbf {X}=\mathbf {x}\mid \theta )f(\theta )}{\int _\mathbb {R} P(\mathbf {X}=\mathbf {x}\mid \theta )f(\theta )\text {d}\theta } \text {d}\theta \nonumber \\&= -\ln \int _\mathbb {R}\frac{g(\theta ) }{f(\theta )} f(\theta \mid \mathbf {X}=\mathbf {x})\text {d}\theta \nonumber \\&\le -\int _\mathbb {R}\ln \frac{g(\theta ) }{f(\theta )} f(\theta \mid \mathbf {X}=\mathbf {x})\text {d}\theta = \int _\mathbb {R}\ln \frac{f(\theta ) }{g(\theta )} f(\theta \mid \mathbf {X}=\mathbf {x})\text {d}\theta \end{aligned}$$

using Jensen’s inequality in the last line. Taking expectations w.r.t. $P_f(\mathbf {X})$ gives the inequality in (2). Similarly, we obtain

$$\begin{aligned} \forall \mathbf {x}: \ln \frac{P(\mathbf {X}_f=\mathbf {x})}{P(\mathbf {X}_g=\mathbf {x})} = \ln \frac{\int _\mathbb {R} P(\mathbf {X}=\mathbf {x} \mid \theta )f(\theta )\text {d}\theta }{\int _\mathbb {R} P(\mathbf {X}=\mathbf {x}\mid \theta )g(\theta )\text {d}\theta } = \ln \int _\mathbb {R}\frac{f(\theta ) }{g(\theta )} g(\theta \mid \mathbf {X}=\mathbf {x})\text {d}\theta , \end{aligned}$$

such that

4 $$\begin{aligned} -\ln \int _\mathbb {R}\frac{g(\theta ) }{f(\theta )} f(\theta \mid \mathbf {X}=\mathbf {x})\text {d}\theta&= \ln \int _\mathbb {R}\frac{f(\theta ) }{g(\theta )} g(\theta \mid \mathbf {X}=\mathbf {x})\text {d}\theta \nonumber \\&\le \int _\mathbb {R}\ln \frac{f(\theta ) }{g(\theta )} f(\theta \mid \mathbf {X}=\mathbf {x})\text {d}\theta . \end{aligned}$$

Since *f* is absolutely continuous w.r.t. *g*, we obtain that both $\frac{f(\theta ) }{g(\theta )}$ and $\ln \frac{f(\theta ) }{g(\theta )}$ are uniformly integrable. Convergence in probability of both posteriors (w.r.t. *f* and *g* as prior) is then sufficient to guarantee the equality in (3) (e.g., Venkatesh, 2013, pp. 480–481), since under these conditions we may change the order of limits and integration.$\square$

The Convergence Theorem relies on posterior consistency. The regularity conditions that imply posterior consistency can be found in many places. For unidimensional monotone IRT models, the regularity conditions for strong consistency (i.e., almost sure convergence) can be found in Chang and Stout (1993, pp. 42–43). As a courtesy to the reader, we list their conditions in Appendix 1. Chang and Stout (1993, pp. 43–45) argued that in practice these conditions are “*very general and appropriate hypotheses*” (p. 51). Similar conditions can be found in Chang (1996) for polytomous IRT models.

Combining Theorem 1, the Monotonicity Theorem, and the Convergence Theorem, we arrive at our final result.

### **Theorem 4**

(Monotone Convergence Theorem) Given an IRT model $P(\mathbf {X} \mid \theta )$ and assuming that the support of *g* contains the support of *f* and the sequence of posteriors converges to a degenerate distribution, then $\Delta (\Theta _f{\text { ; }}\Theta _{\tilde{g}}) \rightarrow 0$, monotonically, and furthermore, $\Theta _{\tilde{g}} \overset{\mathcal {L}}{\longrightarrow }\Theta _f$.

### *Proof*

Under the stated assumptions, the Convergence Theorem implies that the EKL divergence converges to zero as *n* tends to infinity. Convergence is monotone by Theorem 2. From Theorem 1, we consequently obtain

$$\begin{aligned} \Delta (\Theta _f\text { ; }\Theta _{\tilde{g}}) \rightarrow 0 . \end{aligned}$$

Since convergence in KL divergence implies convergence in law (DasGupta, 2008, p. 21), we have

$$\begin{aligned} \Theta _{\tilde{g}} \overset{\mathcal {L}}{\longrightarrow }\Theta _f. \end{aligned}$$

$$\square $$

In summary, the Monotone Convergence Theorem states that (under mild regularity conditions) the marginal distribution of PVs $\tilde{g}$ is a *consistent* estimator of the true ability distribution *f*.

## Implications

In plain words, the Monotone Convergence Theorem implies that we can use PVs to learn about the true distribution of ability. In this section, we discuss some of the practical implications of this result using PISA data for illustration. We remind the reader that *g* is a prior distribution, *f* the true distribution, and $\tilde{g}$ the marginal distribution of the PVs.

### What can we learn from Plausible Values?

What can we learn about the “correct” population model $f(\theta )$ when we are using PVs from the “wrong” posterior $g(\theta \mid \mathbf {X}=\mathbf {x})$? A common misconception is that the marginal distribution of PVs equals the population model (i.e., $\tilde{g} = g$) and nothing can be learned from PVs over that which is already known from the population model (prior distribution) (e.g., Kreiner & Christensen, 2014). This is true, if and only if, the population model is the true ability distribution (i.e., $g = f$). This is not likely and in practice we expect to see that $\tilde{g} \ne g$.

#### *Example 2*

**Fig. 2:**
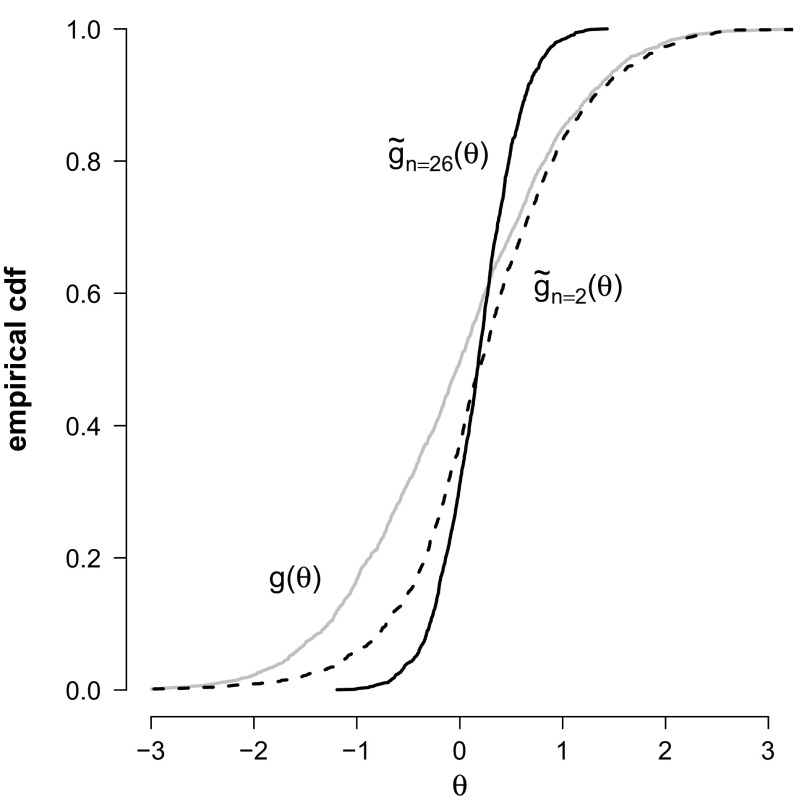
Ecdf of PVs ($\tilde{g}$) and *N* draws from a standard normal prior distribution (i.e., $g(\theta )=\phi (\theta )$) in the PISA example.

(PISA) To illustrate that the PV distribution may diverge from the prior in applications, we analyze data from the 2006 PISA cycle. More specifically, we used the $n = 26$ items intended to assess reading ability in booklet 6 made by $N =$ 1738 Canadian students (see Appendix 2 for details of this analysis). A single PV was generated for each student using the *One Parameter Logistic Model* (OPLM; Verhelst & Glas, 1995) as IRT model, and a standard normal distribution as prior. The ecdf of *N* draws from the prior distribution *g* (solid gray line) and the ecdf of the generated PVs using $n=26$ items are shown in Fig. 2 (solid black line). The marginal distribution of the PVs is clearly different from the specified prior distribution.

If the population model is misspecified (i.e., $g \ne f$), we can still learn a lot from looking at the PV distribution. The PV distribution provides a consistent estimate of the true ability distribution, which is at least as plausible as the population model which figures as a prior. Specifically, it follows from the Monotonicity Theorem that, if $g \ne f$, and hence $\tilde{g} \ne g$, the marginal distribution of PVs $\tilde{g}$ is closer to *f* than *g* is; as we saw in Example 1. Moreover, we can use PVs to evaluate the fit of the population model by testing the hypothesis $H_0 : \tilde{g} = g$ against $H_1 : \tilde{g} \ne g$. If $H_0$ is rejected, there is no reason to be interested in *g*: $\tilde{g}$ is our best guess of what the true distribution of ability would look like.

#### *Example 3*

(PISA continued) We use the PISA example to illustrate that we can test the hypothesis $H_0: \tilde{g} = g$ against $H_1: \tilde{g} \ne g$ using real data with a relatively small number of observations, and that the power of this test is increasing with *n*. To this aim, we randomly assigned each student two items out of the 26 items that were available. Figure 2 shows the ecdf of the PVs using $n = 2$ items (dashed line). It is clear that even with two items, the marginal distribution of PVs differs from the specified prior distribution and $H_0$ does not hold (this test is performed in the next example, see Table 1). Figure 2 also shows that the PV distributions diverge from the prior distribution as *n* increases, thereby increasing the probability to reject $H_0$ if it is wrong.

**Table 1:** Average values of KS test statistic using PISA data to compare $\tilde{g}$ with the prior distributions used to generate $\tilde{g}$.

	$$g(\theta )$$		
*n*	$\mathcal {N}(0\text {, }1)$	$\mathcal {N}(\mu \text {, }\sigma ^2)$	$\mathcal {N}(\widehat{\varvec{\Lambda }}\varvec{\beta }\text {, }\sigma ^2)$
26	0.292	0.034	0.026
20	0.290	0.032	0.026
14	0.277	0.032	0.026
8	0.256	0.030	0.026
2	0.161	0.029	0.028

Values over 0.046 are significant at an ${\alpha }$ level of 0.05.

### Choose a flexible population model

The population model is formally a prior and, under the conditions of the Monotone Convergence Theorem, becomes irrelevant as the number of items becomes large. Essentially, this is an instance of the common finding that the data overrule the prior when the number of observations increases. In practice, however, there is a natural limit to the number of items that can be administered which raises the question how we can favor convergence without increasing the number of items.

The answer comes from Lemma 1 which suggests that convergence of the PV distribution to the true distribution of ability is faster if prior divergence is reduced. Thus, for a given *n*, we would like prior divergence to be as small as possible (i.e., we would like *g* to resemble *f*). When little or nothing is known about *f*, we may achieve this using a *flexible prior*; that is, one that easily adapts to different shapes. Otherwise, we may look at the PV distribution found in previous editions of the study to improve the prior. Convergence is also improved if we adopt an empirical Bayesian approach and estimate the parameters of the prior so that it adapts itself to the data as much as possible (see, for instance; White, 1982). Using, for instance, a normal prior in Example 1 would help to discover the bimodality of the true ability distribution with less items.

#### *Example 4*

(PISA Continued) We use the previously established OPLM model with three prior distributions ordered in terms of flexibility:

1. A standard normal distribution $\mathcal {N}(0\text {, }1)$.
2. A normal distribution $\mathcal {N}(\mu \text {, }\sigma ^2)$ with mean $\mu$ and variance $\sigma ^2$.
3. A PCA regression prior $\mathcal {N}(\widehat{\varvec{\Lambda }}\varvec{\beta }\text {, }\sigma ^2)$, where $\widehat{\varvec{\Lambda }}$ constitutes the principal component scores estimated on student covariates assessed in the PISA student questionnaire. We use the first 50 principal components explaining roughly $60~\%$ of the variance in the student questionnaire.

The parameters of the prior distribution are estimated using the Gibbs sampler (Geman & Geman, 1984) with non-informative hyper-priors (Gelman, Carlin, Stern, & Rubin, 2004).

For each prior distribution, we test the hypothesis $H_0 : \tilde{g} = g$ against $H_1 : \tilde{g} \ne g$ using the two-sample Kolmogorov-Smirnov (KS) test. For the second and third prior, we ran an additional 1000 iterations of the Gibbs sampler. In each iteration, we generated one PV for each person, generate a sample of size *N* from the prior, and compute the KS test statistic. Thus, we obtained 1000 replications for the test statistic, which were then averaged. The results are shown in Table 1 and confirm that prior divergence decreases as more flexible prior distributions are used.

Our main concern is whether or not the PV distribution converges to the true ability distribution. Since we do not know the true ability distribution, we compare our results with the best guess that we have, i.e., the distribution of PVs obtained by using $n = 26$ items and the PCA regression prior. We repeated the procedure to obtain Table 1, but instead of comparing the generated PVs with draws from the prior, we compared the generated PVs with the PVs generated using $n = 26$ items and the PCA regression prior. The results in Table 2 show that the PV distributions converge to a single (true) distribution as *n* increases and/or the prior becomes more flexible.

**Table 2:** Average values of KS test statistic using PISA data to compare $\tilde{g}$ using different prior distributions with the best guess.

	$$g(\theta )$$		
*n*	$\mathcal {N}(0\text {, }1)$	$\mathcal {N}(\mu \text {, }\sigma ^2)$	$\mathcal {N}(\widehat{\varvec{\Lambda }}\varvec{\beta }\text {, }\sigma ^2)$
26	0.052	0.021	–
20	0.064	0.022	0.022
14	0.088	0.024	0.024
8	0.133	0.030	0.027
2	0.235	0.065	0.109

It is important to note that there is a limit to the amount of parameters that we can estimate, and thus the amount of flexibility that we can achieve in practice. This can be seen in Example 4. For $n=2$, Table 2 seems to suggest that the normal prior works better than the more flexible PCA regression prior. This counter intuitive result only holds for $n=2$ and is due to the poor estimation of hyper-parameters that results when both *N* and *n* are small. The normal prior has just two parameters, $\mu$ and $\sigma ^2$, whereas the PCA regression prior has 52 parameters, $\varvec{\beta } = \{\beta _0\text {, }\beta _1\text {, }...\text {, }\beta _{50}\}$ and $\sigma ^2$. Since the standard errors accumulate for the generated PV distributions, we expect to observe larger variations in the generated PV distributions using the PCA regression prior. These larger variations are reflected in the value of the KS test statistic.

### What if we miss a covariate?

A remarkable feature of Example 1 is that the PV distribution reveals the difference between boys and girls even though sex was not included as a covariate in the population model. This is consistent with our results. Given the conditions of the Monotone Convergence Theorem, the distribution of plausible values

$$\begin{aligned} \tilde{g}(\theta \mid z_1\text {, } z_2)=\sum _{\mathbf {x}} g(\theta \mid \mathbf {x}\text {, }z_2)P_f(\mathbf {x}\mid z_1\text {, }z_2) \end{aligned}$$

is a consistent estimator of the population distribution $f(\theta \mid z_1\text {, }z_2)$, for sets of covariates $z_1$ and $z_2$; even when $z_2$ is the empty set (i.e., if we miss all covariates). It also means that a secondary analyst who happens to observe the student’s sex will, when *n* is sufficiently large, recover the difference between boy and girls even if the PVs have been generated with a population model that contains no covariates at all.

**Fig. 3:**
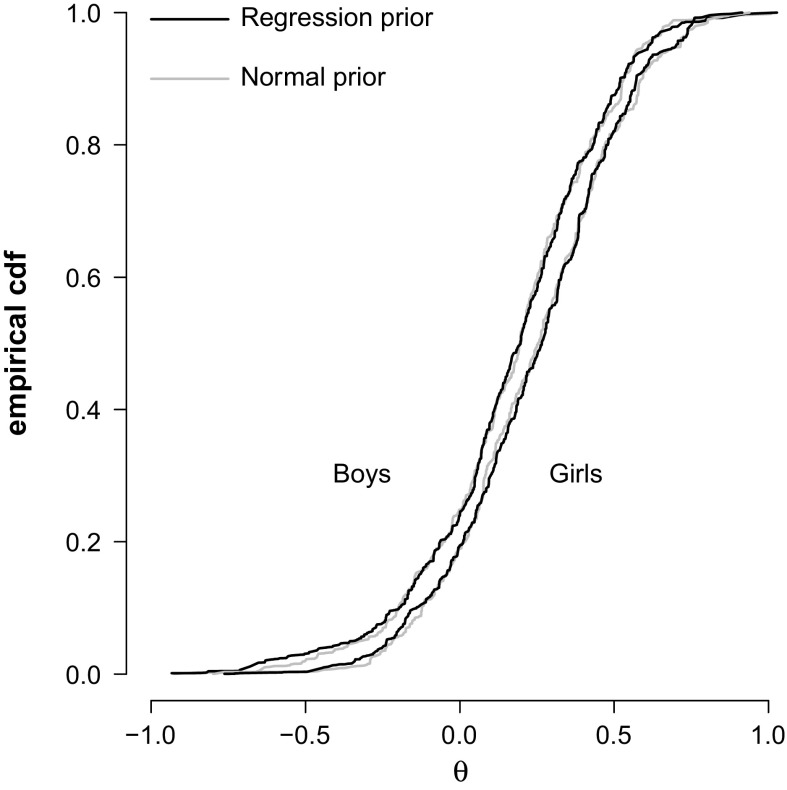
Plausible value distributions of boys and girls with and without gender as a covariate in the PISA example.

#### *Example 5*

(PISA Continued) We look at the distribution of boys and girls in Canada who took booklet 6 using PISA’s final student weights. To generate the PVs we consider two prior distributions; the flexible $\mathcal {N}(\mu \text {, }\sigma ^2)$ prior distribution without covariates, and the $\mathcal {N}(\widehat{\varvec{\Lambda }}\varvec{\beta }\text {, }\sigma ^2)$ prior distribution which included gender as a predictor (i.e., it was a covariate in the PCA).

Figure 3 shows the PV distributions of boys and girls weighted by the PISA student weights. It is clear that the weighted distributions of PVs under the two prior distributions are indistinguishable, apart from sampling error. We also see that the girls perform better than the boys. The weighted average ability for the boys was estimated at 0.180 and that of girls at 0.242. The weighted standard deviation of ability for the boys was estimated at 0.304 and that of the girls at 0.282. Note that the differences in variances between boys and girls would not have been found in a latent regression model unless it had been explicitly modeled.

What it means for *n* to be “sufficiently large” depends on the effect of the covariate on the distribution of $\Theta$; that is, for large effects relatively many items are needed, and for small effects relatively few items are needed. It also depends on the population model. Institutions that release PVs typically include a large set of covariates in the population model on the argument that any covariate that a secondary analyst might be interested in must be included, directly or by proxy, to avoid bias in secondary analysis of the PVs. Schofield, Junker, Taylor, and Black (2015) make this claim precise and, in accordance with our results, argue that bias should vanish when $n \rightarrow \infty$. We agree to the current practice to include as many covariates as possible because it reduces prior divergence but note that a flexible prior with or without covariates can be used to the same effect. A simple extension of Example 1 would illustrate, for instance, that, if a binary predictor is excluded from the population model, the correct coefficient will be recovered even for small *n* when the prior distribution is a mixture of two normal distributions.

If the population model is a regression model in which a covariate is missing, this may not only lead to bias in the PV distributions but may also lead to bias in parameter estimates for effects that are part of the model^3^, or one might not observe that the missing covariate makes the unknown *f* skewed. This means that we run the risk of performing an incorrect inference about the unknown *f* if we look at the population model. It follows from our results that the marginal distribution of the PVs will always be a better estimate of *f* than the population model is in this situation, even if we do not recover the correct regression coefficient of the missing covariate.

## Discussion

In this paper, we have proved that, under mild regularity conditions, the empirical distribution of the PVs is a consistent estimator of the distribution of ability in the population, and that convergence is *monotone* in an embedding in which the number of items tends to infinity. In plain words, this implies that we can use PVs to learn about the true distribution of ability in the population. We have used this result to clear up some of the misconceptions about PVs, and also to show how they can be used in the analyses of educational surveys. Thus far, PVs have been used in educational surveys mostly to simplify secondary analyses. Our result suggests that the distribution of PVs could play the leading role, using the population model merely as a vehicle to produce PVs.

The population model is properly seen as a prior and the consistency of the PV distribution as an estimator of the true distribution is essentially the common result that the data overrule the prior when the number of observations increases. We have demonstrated that convergence of the PV distribution to the true distribution of ability can be improved if we estimate the parameters $\varvec{\lambda }$ of the prior distribution, but it does not imply that it makes sense to interpret the estimates $\widehat{\varvec{\lambda }}$ when the prior distribution is misspecified. Technically, as the number of persons in the sample, *N*, tends to infinity, $\widehat{\varvec{\lambda }}$ are the parameter values that minimize prior divergence under the prior w.r.t. the true ability distribution (White, 1982). However, when the prior distribution is misspecified and prior divergence is not zero, the result of White (1982) does not tell us how wrong our conclusions are when inference is based on $\widehat{\varvec{\lambda }}$.

In closing, we mention a limitation of our results. Our results imply that *if* the sequence of posteriors converges to a degenerate distribution as *n* tends to infinity, *then* the marginal distribution of PVs converges to the unknown *f*. For models where the “if" part is resolved, our results (i.e., Theorems 3, 4) apply.

## Appendix 1: The regularity conditions in Chang and Stout (1993)

In order to prove their Theorem 2, Chang and Stout (1993) require five regularity conditions. Before we give these conditions, we need to fix some notation. Let $X_i$ denote the response of a person to an item *i*, where $X_i = 1$ denotes a correct response and $X_i=0$ an incorrect response, where

5 $$\begin{aligned} X_i = {\left\{ \begin{array}{ll} 1 &{} \text { with probability } P_i(\theta ) = P(X_i = 1 \mid \theta ),\\ 0 &{} \text { with probability } 1-P_i(\theta ) = P(X_i = 0\mid \theta ), \end{array}\right. } \end{aligned}$$

where $P_i(\theta )$ denotes the probability of a correct response for a person with ability $\theta$, and $\theta$ is unknown and has the domain $(-\infty \text {, }\infty )$ or some subinterval thereof. Chang and Stout (1993, p. 38) made two assumptions about the unidimensional IRT model:

1. Local independence:
  $$\begin{aligned} P(X_1 = x_1\text {, }\ldots \text {, }X_n = x_n\mid \theta ) = \prod _{i=1}^n P_i(\theta )^{x_i}(1-P_i(\theta ))^{1-x_i}. \end{aligned}$$
2. Monotonicity: each $P_i(\theta )$ is strictly increasing in $\theta$.

Note that these conditions are standard assumptions in parametric unidimensional IRT models, and are satisfied for the commonly used One-, Two- and Three-parameter logistic and normal ogive models.

Fix any $\theta _0 \in \Theta$ (the latent space), then the five regularity conditions are as follows (Chang & Stout, 1993, pp. 42–43):

1. Let $\theta \in \Theta$, where $\Theta$ is $(-\infty \text {, }\infty )$ or a bounded or unbounded interval of $(-\infty \text {, }\infty )$. Let the prior density $f(\theta )$ be continuous and positive at $\theta _0$, where $\theta _0$ is assumed to be the true value of $\theta$.
2. $P_i(\theta )$ is twice continously differentiable and $P_i^{\prime }(\theta )$ and $P_i^{\prime \prime }(\theta )$ are bounded in absolute value uniformly with respect to both $\theta$ and *i* in some closed interval $N_0$ of $\theta _0 \in \Theta$.
3. For every fixed $\theta \ne \theta _0$, assume for some given $c(\theta ) > 0$,
  $$\begin{aligned} \overline{\lim _{n\rightarrow \infty }} \text { }\frac{1}{n} \sum _{i=1}^n \mathbb {E}_{\theta _0}\left( \frac{P_i(\theta )^{X_i}(1-P_i(\theta )^{1-X_i}}{P_i(\theta _0)^{X_i}(1-P_i(\theta _0)^{1-X_i}}\right) \le -c(\theta ), \end{aligned}$$
  and
  $$\begin{aligned} \sup _i\left| \lambda _i(\theta )\right| = \sup _i\left| \log \left( \frac{P_i(\theta )}{1-P_i(\theta )}\right) \right| < \infty . \end{aligned}$$
  (For a sequence of real numbers $\{a_n\}$, if $\lim _{n\rightarrow \infty } a_n$ does not exist, then $\{a_n\}$ must have more than one limit point. In this case, $\overline{\lim _{n\rightarrow \infty }} \text { }a_n$ denotes the largest such or upper limit point. Also, $\mathbb {E}_{\theta _0}(W)$ denotes the expectation of *W* with $\theta = \theta _0$ assumed.)
4. $\{I^{\prime }_i(\theta )\}$ and $\{\lambda _i^{\prime }(\theta )\}$ and $\{\lambda _i^{\prime \prime }(\theta )\}$ are bounded in absolute value uniformly in *i* and $\theta \in N_0$, where $N_0$ is specified in (A2) above.
5. $$\begin{aligned} \liminf _{n \rightarrow \infty } \frac{1}{n} I^{(n)}(\theta _0) > c(\theta _0) > 0. \end{aligned}$$
  That is, asymptotically, the average information at $\theta _0$ is bounded away from 0.

Note that we have used $f(\theta )$ to denote the prior density and *i* to index the items, whereas Chang and Stout (1993) used $\Pi (\theta )$ and *j*, respectively, in their manuscript.

## Appendix 2: Details about the PISA analyses

We used item response data from Booklet 6 in the 2006 PISA cycle. Specifically, we used the responses from $N = 1768$ Canadian students to $n = 28$ items intended to assess reading ability. The data of 30 students were omitted due to missing responses, and we fitted a *One Parameter Logistic Model* (OPLM; Verhelst & Glas, 1995) on data from the remaining $N = 1738$ students.

The item difficulties were estimated using *conditional maximum likelihood* and the item discriminations were estimated using *marginal maximum likelihood* using the OPLM package (Verhelst, Glas, & Verstralen, 1995). We used cross-validation for estimation of the (discrete) item discriminations; First, the discriminations were estimated based on data from a random selection of 1200 students. At this stage, we deleted two items that did not fit the scale (items 6 and 8). The remaining $n = 26$ items scaled reasonably well in this sample, $R_{1C}= 133.067$, $df =90$, $p = 0.0022$ (for a description of the $R_{1C}$ statistic see Verhelst et al., 1995). Second, the parameters were validated on data from the remaining 538 students, and scaled well, $R_{1C}= 118.686, df =90, p=0.0231$.

The estimated item parameters are shown in Table 3, where *a* indicates item discrimination and *b* item difficulty (category thresholds for polytomous items). For polytomous items, score categories are indicated within parentheses after the item number.

**Table 3:** Parameters of the estimated IRT model for the PISA example.

Item (cat.)	*a*	*b*	Item (cat.)	*a*	*b*
1	3	$-$0.603	15(1)	3	$-$0.092
2	3	$-$0.035	15(2)	3	0.442
3	5	$-$0.089	16(1)	3	0.299
4	5	$-$0.215	16(2)	3	$-$0.093
5	4	$-$0.428	17	4	$-$0.061
6	$-$	$-$	18	5	$-$0.163
7(1)	3	0.252	19	5	$-$0.442
7(2)	3	$-$0.466	20	5	0.269
8	$-$	$-$	21	5	0.047
9	4	0.173	22	5	0.135
10	5	$-$0.390	23	5	$-$0.149
11	4	$-$0.334	24	3	0.065
12	3	0.481	25	2	0.084
13(1)	4	0.316	26(1)	3	$-$0.140
13(2)	4	0.871	26(1)	3	0.400
14	5	$-$0.041	27	6	0.072
			28	6	$-$0.167

## Footnotes

1 The prior *g* usually conditions on a large set of covariates $\mathbf {Z}$ and in the parlance of educational surveys is known as the population or *conditioning model* for the survey. To avoid excessive notation, we will present the main results without explicitly mentioning the conditioning on covariates.

2 We assume in this paper that we obtain a simple random sample from $P(\mathbf {X}_f)$ (i.e., $f(\theta )$). In educational surveys, one typically obtains non-simple random samples. We note that our results generalize to the latter situation.

3 The simplest example would be a prior where the mean is assumed to be equal to zero and one estimates the variance. If the true mean is not equal to zero, the variance estimate will be biased.
